# Supplementary material for: RNA structures within Venezuelan equine encephalitis virus E1 alter macrophage replication fitness and contribute to viral emergence
Source: bioRxiv. 2024 Apr 9:2024.04.09.588743. Preprint. [Version 1] doi: 10.1101/2024.04.09.588743 (PMC11030350; doi:10.1101/2024.04.09.588743)
Supplement: Supplement 1 [file NIHPP2024.04.09.588743v1-supplement-1.pdf]

bioRxiv preprint doi: https://doi.org/10.1101/2024.04.09.588743; this version posted April 9, 2024. The copyright holder for this preprint (which was not certified by peer review) is the author/funder, who has granted bioRxiv a license to display the preprint in perpetuity. It is made available under aCC-BY 4.0 International license.

Table S1: Primer sequences for generation of RIG-I and MDA5 CRISPR cell lines

Gene	Primer	Sequence
Ddx58	Ddx58.g19.F	5'- CACCGAAGAACAACAAGGGCCCAA-3'
	Ddx58.g19.R	5'- AAACTTGGGCCCTTGTTGTTCTTC-3'
	Ddx58.g28.F	5'- CACCGATATCATTTGGATCAACTG-3'
	Ddx58.g28.R	5'- AAACCAGTTGATCCAAATGATATC-3',
	Ddx58.g36.F	5'- CACCGTGGATTGTTGATAAAGGTG-3'
	Ddx58.g36.R	5'- AAACCACCTTTATCAACAATCCAC-3'
Ifih1	Ifih1.g24.F	5'-CACCGTGTGGGTTTGACATAGCGCG-3'
	Ifih1.g24.R	5'-AAACCGCGCTATGTCAAACCCACAC-3'

bioRxiv preprint doi: https://doi.org/10.1101/2024.04.09.588743; this version posted April 9, 2024. The copyright holder for this preprint (which was not certified by peer review) is the author/funder, who has granted bioRxiv a license to display the preprint in perpetuity. It is made available under aCC-BY 4.0 International license.

	Ifih1.g69.F	5’-CACCGTTGGCGCAGAACATCCAGGA-3’
	Ifih1.g69.R	5-‘AAACTCCTGGATGTTCTGCGCCAAC-3’
	Ifih1.g81.F	5’-CACCGCGTAGACGACATATTACCAG-3’
	Ifih1.g81.R	5’-AAACCTGGTAATATGTCGTCTACGC-3’

Table S2: KC344519 E1 gene block

CGACCACGATGCCGAGCCAAGCGGGAATCTCGTATAACACCATAGTCAACAGAGCAGGCTACGCGCCACTCCCTATCAGCATAACACCAACAAAGATCAAGCTGATACCCACAGTGAACTTGGAGTACGTCACCTGCCATTACAAAACAGGAATGGATTCACCAGCCATCAAATGCTGCGGATCTCAGGAATGCACTCCAACTTACAGGCCCGATGAACAGTGCAAAGTCTTCACAGGGGTTTACCCGTTCATGTGGGGAGGTGCATATTGCTTTTGCGACACTGAGAACACCCAAGTCAGCAAGGCCTACGTAATGAAATCTGACGACTGCCTTGCGGATCACGCTGAAGCATACAAAGCGCACACAGCCTCAGTGCAGGCATTCCTCAACATCACAGTGGGAGAACACTCCATTGTGACCACCGTGTACGTGAATGGAGAAACTCCTGTGAACTTCAATGGGGTCAAAATAACTGCAGGTCCACTTTCCACAGCTTGGACACCCTTTGACCGCAAAATCGTGCAGTATGCCGGGGAGATCTATAATTACGATTTTCCTGAGTATGGGGCAGGACAACCAGGAGCATTCGGAGACATACAATCCAGAACAGTCTCAAGCTCAGATCTGTATGCCAATACCAACCTAGTGCTGCAGAGACCCAAAGCAGGAGCGATCCATGTGCCATACACTCAGGCACCATCGGGTTTTGAGCAATGGAAGAAAGATAAAGCTCCGTCATTGAAATTCACCGCCCCTTTCGGATGCGAAATATATACAAACCCCATTCGCGCCGAAAATTGTGCTGTAGGGTCAATTCCATTAGCCTTTGACATCCCTGACGCCCTGTTCACCAGGGTGTCAGAAACACCGACACTTTCAGCGGCCGAATGTACTCTTAATGAGTGCGTGTATTCATCCGACTTTGGCGGGATCGCCACGGTCAAGTATTCGGCCAGCAAGTCAGGCAAGTGCGCAGTCCATGTGCCATCAGGGACTGCTACCCTAAAAGAAGCAGCAGTCGAGCTAACCGAGCAAGGGTCGGCGACCATTCATTTCTCGACCGCAAATATCCACCCGGAGTTCAGGCTCCAAATATGCACATCATATGTCACGTGCAAAGGTGATTGTCACCCCCCGAAAGACCACATTGTGACACACCCCCAGTATCACGCCCAAACATTCACAGCCGCGGTGTCAAAAACCGCGTGG

bioRxiv preprint doi: https://doi.org/10.1101/2024.04.09.588743; this version posted April 9, 2024. The copyright holder for this preprint (which was not certified by peer review) is the author/funder, who has granted bioRxiv a license to display the preprint in perpetuity. It is made available under aCC-BY 4.0 International license.

ACGTGGTTAACATCCCTGCTGGGAGGATCG**GCCGTAATTATTATAATTGGCTTGGTGCTG GCTACTATTGTGGCCATGTACGTGCTGACC**

Table S3 DsiRNA sequences

Gene	**duplex name**	**DsiRNA sequence**
Ehmt2	mm.Ri.Ehmt2.13.1	AUCAAUGCAGUAAACCUCGCCAUCCUU
	mm.Ri.Ehmt2.13.2	GGUAAGAAUCAUCCUCUCUCACAUCAG
	mm.Ri.Ehmt2.13.3	UACCAAACCCAACAUUUAUUGAGAACA
Zc3h4	mm.Ri.Zc3h4.13.1	ACAAUUUAUGACUCAAAGAAAGUACUA
	mm.Ri.Zc3h4.13.2	UAUAAUUUACACGGAAAGUCACCAUGC
	mm.Ri.Zc3h4.13.3	UGUCCUUGGACCUGCGGUACUGGUUCA
Thrap3	mm.Ri.Thrap3.13.1	UUUAGUAAAGCGUUCAUGCAAUGUCAU
	mm.Ri.Thrap3.13.2	GUCGUCAUGCAAGUAAUACUUCUUGCU
	mm.Ri.Thrap3.13.3	AAUUCAGAAGUUUCUUAGAAAACCGUC
Syncrip	mm.Ri.Syncrip.13.1	CUGAAGUAGUAUCCAUGGGCUCUUCAG
	mm.Ri.Syncrip.13.2	AGUAGUAAUCAUCAUACAUUUGAUUCU
	mm.Ri.Syncrip.13.3	GAAUGAAAGCAUAAUCUUUUAGCUUCU
Bclaf1	mm.Ri.Bclaf1.13.1	UGACCUUGGACUAUUAUCAUAAGCUGA
	mm.Ri.Bclaf1.13.2	UAUGUAAGCUAUCCAAUCAGGUACUAG
	mm.Ri.Bclaf1.13.3	ACUCUUUAUCACUAAAGUAAUCUAGAA
Fbl	mm.Ri.Fbl.13.1	CAGAUAAAGACACCUUCAUGACGAUGC
	mm.Ri.Fbl.13.2	CAGGAAUAAUGUUAGUCCUCUUCUUGG
	mm.Ri.Fbl.13.3	UCUGCUGACGCAGUGGAGUCAAUGCAG
Chtop	mm.Ri.Chtop.13.1	AACAUUUCCAAUUUACUACCUUCACUA

bioRxiv preprint doi: https://doi.org/10.1101/2024.04.09.588743; this version posted April 9, 2024. The copyright holder for this preprint (which was not certified by peer review) is the author/funder, who has granted bioRxiv a license to display the preprint in perpetuity. It is made available under aCC-BY 4.0 International license.

	mm.Ri.Chtop.13.2	UUUAGUUUUCGACAUGUAUGCAUCCAA
	mm.Ri.Chtop.13.3	CUGCUUAAGUUUUAAUGCUGCCUGGAC
Ubap2l	mm.Ri.Ubap2l.13.1	CCUGUUGUACUGCCACCUUUAGCUUCC
	mm.Ri.Ubap2l.13.2	AAGUGGAAGGUGAUUCAGAUUUCACUG
	mm.Ri.Ubap2l.13.3	CUGUGAUAUCAAUCAAUUGUUUCACCU
Dhx38	mm.Ri.Dhx38.13.1	AUCUUCAUGCAAGUACUGGGUCAGCUG
	mm.Ri.Dhx38.13.2	ACAGGUGUACCUGCAAGGACUGCUUCA
	mm.Ri.Dhx38.13.3	ACAGAUGAACACGCAGGUGAAAGCCUU
Hnrnpr	mm.Ri.Hnrnpr.13.1	GUCAUUGUACUCUAUAUAGGUUUAACC
	mm.Ri.Hnrnpr.13.2	AACCUAGGUAAGGUUUCUUAUUUUGAU
	mm.Ri.Hnrnpr.13.3	AAUUCUUGGUUGUCAUUAUUGUAACCA
Ubtf	mm.Ri.Ubtf.13.1	UACCGUACUCGGAAUUUCGAAAAGAGA
	mm.Ri.Ubtf.13.2	CUCCAUUCCUUCAAGGCAUGUAACUGU
	mm.Ri.Ubtf.13.3	UUUGUAAGGGUUUUUAACAUGUUCCUG
Ddx58	mm.Ri.Ddx58.13.1	CACCUAACAACUAUUUCCAAAGUUUUC
	mm.Ri.Ddx58.13.2	GAUCUUCAAUGAACAUUAAGUAGUCAA
	mm.Ri.Ddx58.13.3	AAGCUCUAAGGUCAGUAUUUUUAACUU
Ifih1	mm.Ri.Ifih1.13.1	ACCUACUCCGUAGAAUAGCUAUUCGUC
	mm.Ri.Ifih1.13.2	AGGCUCUCUUCUACUACAUAUUUCGAU
	mm.Ri.Ifih1.13.3	GUUGUAGUACUCUGCUAUAAACUUCGU
Mavs	mm.Ri.Mavs.13.1	ACGUAUGUAACUACGAUUAUAUAAGAU
	mm.Ri.Mavs.13.2	UCUAACCAGGGUCAUUUUUGGUACAGA
	mm.Ri.Mavs.13.3	UGGACAAAGUCAUGAUGUGAGUGGUUA
